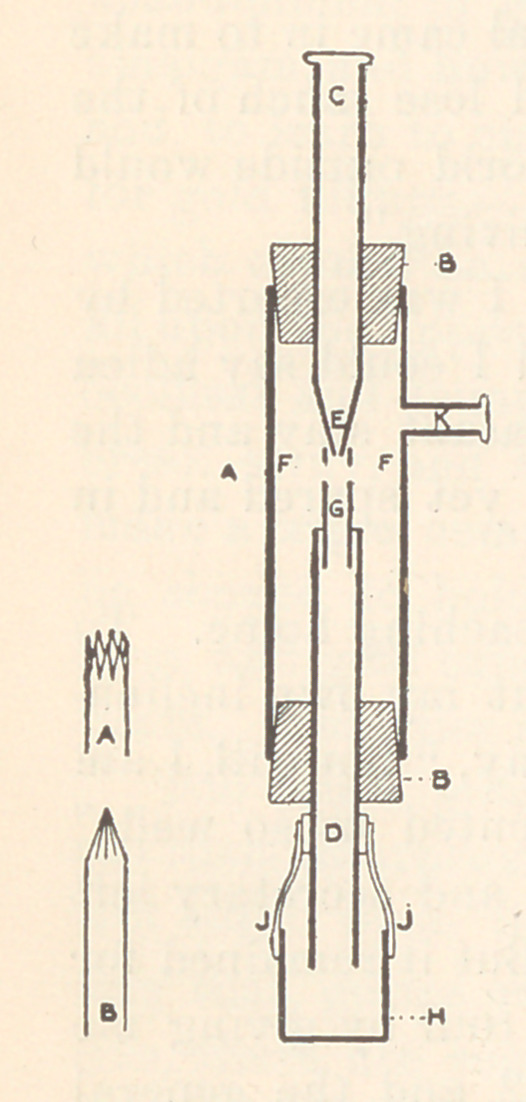# A Simple Saliva Ejector

**Published:** 1898-12

**Authors:** J. J. H. Sanders


### Abstracts and Translations.

### A SIMPLE SALIVA EJECTOR.

BY J. J. H. SANDERS, L.D.S.I.

THE saliva ejector has become such a necessity in the every-day life of the dentist that no operating-room can be considered properly equipped without one. The instrument described below makes no claim to elaborate appearance, but it can be depended on to do its work, and has the merit of being both easy and cheap to construct.

In its simplest form it can be easily made in about one hour by any one who can soft-solder, and I think the following particulars and drawing will make its construction clear.

Before describing the ejector it may be well to give a rough outline of its action, so as to render the details more intelligible.

Referring to the sectional drawing, it will be seen that the ejector consists of the tube A, forming the body of the instrument, the ends of which are closed by two corks, B, B. Through these pass two smaller tubes, C, D, the upper one terminating in the fine jet E, and the lower having a short piece of smaller tube soldered in it, as at G. The upper tube C is attached to a water-supply, and a fine stream issues from the jet. This, in rushing through the narrow tube G, carries with it some of the air, thus creating a partial vacuum, to restore which air passes through the small holes (shown at F, F) drilled in the tube. In order that the vacuum may be formed and maintained in the throat (G) it is essential that the end of the tube D should dip under water, otherwise air would pass up the tube, and no vacuum could be formed. This condition is obtained by slipping a small bucket over the end of the tube D.

The ejector is best made from brass tubing, and for this purpose fishing-rod ferrules are admirably suited. These may be obtained in all sizes and at a small cost at any fishing-tackle maker.

The body tube A is about three and one-half inches long and three-fourths of an inch in diameter, on the back of which is soldered a plate of brass (not shown in the drawing) for the purpose of screwing the ejector to the wall. A short length of tube, K, is also soldered at a convenient point for attaching the suction-tube.

The corks used for closing the ends of the main tube must be specially selected for their soundness, and may with advantage be dipped in melted wax before using. Two lengths of smaller brass tubing, about one-fourth or five-sixteenths inch, are taken to form the tubes C and D. As will be seen, the upper tube C terminates in the point E, which has a fine aperture to allow a jet of water to pass. Possessors of a turning-lathe will, of course, turn this up, but if one is not available, proceed as follows: A series of cuts are made with a fine saw at the end of the tube and triangular pieces removed, as shown at A, Fig. 2; the remaining pieces are then bent together until they form a point, as at B. Soft solder is then run into the cuts, and the small hole drilled with a fine drill in the engine.

This hole must be quite smooth, and about one-thirtieth of an inch in diameter, and must be so drilled that the jet is thrown in the direction of the axis of the tube. If the ejector is to be attached to the water-supply by rubber tubing, it will be well to solder a ring of wire at the top of the tube to prevent its being blown off by the water-pressure. This finishes the upper tube.

The lower tube D has a short length of small brass tube soldered into its upper end to form the throat, as at G; it should be about four times the diameter of the jet aperture, and about three-fourths of an inch long. It must be soldered centrally into the larger tube D, which may be adapted to receive it in the same manner as the jet was formed.

Two or more holes of about one-sixteenth inch diameter are drilled in the throat at one-eighth inch below its upper edge, as shown at F, F, to permit the air and water to be drawn from the body of the ejector. Before putting the parts together there remains the bucket (H) to be made. For this take a short piece of tube about three-fourths of an inch in diameter and one inch long, and close one end by soldering in a disk of brass. Two narrow strips of brass are then soldered to the other end, as shown at J, J, and these, having been bent to the proper curve, are again soldered to a short piece of split tube which will just slide over the end of the tube D.

The parts may now be put together. First the holes must be bored in the corks to take the tubes C, D, for which purpose a cork-borer should be used if one is at hand. If not, however, the holes may be made by taking a piece of the same tube as C and D are made from and, having sharpened one end with a smooth file, slowly pushing it through the cork with a twisting motion. The corks are now tightly inserted into the main tube, and the tubes C, D pushed through until the jet fits into the throat G; this insures the jet being central with the throat, and gives steadiness to the tubes. The bucket H is pushed on the end of D until the tube nearly touches its bottom, and the ejector is ready for use. It will work best on a high-pressure water-supply, but will also do its work well if supplied from a cistern, provided a fall of four or five feet can be obtained.

The following conditions are necessary for successful working:

1. All joints must be tight.

2. The jet of water must pass through the throat G without touching its walls.

3. The end of the tube D must be under water when working.
—*Journal of the British Dental Association.*

## Figures and Tables

**Figure f1:**